# An interpretable and transparent machine learning framework for appendicitis detection in pediatric patients

**DOI:** 10.1038/s41598-024-75896-y

**Published:** 2024-10-18

**Authors:** Krishnaraj Chadaga, Varada Khanna, Srikanth Prabhu, Niranjana Sampathila, Rajagopala Chadaga, Shashikiran Umakanth, Devadas Bhat, K. S. Swathi, Radhika Kamath

**Affiliations:** 1 Department of Computer Science and Engineering, Manipal Institute of Technology, Manipal Academy of Higher Education, Manipal, 576104 Karnataka India; 2 Department of Biostatistics, Yale School of Public Health, Yale University, New Haven, Connecticut, 06510 USA; 3 Department of Biomedical Engineering, Manipal Institute of Technology, Manipal Academy of Higher Education, Manipal, 576104 Karnataka India; 4 Department of Mechanical and Industrial Engineering, Manipal Institute of Technology, Manipal Academy of Higher Education, Manipal, 576104 Karnataka India; 5 Department of Medicine, Dr. TMA Pai Hospital, Manipal Academy of Higher Education, Manipal, 576104 Karnataka India; 6 Department of Social and Health Innovation, Prasanna School of Public Health, Manipal Academy of Higher Education, Manipal, 576104 Karnataka India

**Keywords:** Diagnostic markers, Predictive markers, Risk factors, Biomedical engineering, Computational science

## Abstract

Appendicitis, an infection and inflammation of the appendix is a prevalent condition in children that requires immediate treatment. Rupture of the appendix may lead to several complications, such as peritonitis and sepsis. Appendicitis is medically diagnosed using urine, blood, and imaging tests. In recent times, Artificial Intelligence and machine learning have been a boon for medicine. Hence, several supervised learning techniques have been utilized in this research to diagnose appendicitis in pediatric patients. Six heterogeneous searching techniques have been used to perform hyperparameter tuning and optimize predictions. These are Bayesian Optimization, Hybrid Bat Algorithm, Hybrid Self-adaptive Bat Algorithm, Firefly Algorithm, Grid Search, and Randomized Search. Further, nine classification metrics were utilized in this study. The Hybrid Bat Algorithm technique performed the best among the above algorithms, with an accuracy of 94% for the customized APPSTACK model. Five explainable artificial intelligence techniques have been tested to interpret the results made by the classifiers. According to the explainers, length of stay, means vermiform appendix detected on ultrasonography, white blood cells, and appendix diameter were the most crucial markers in detecting appendicitis. The proposed system can be used in hospitals for an early/quick diagnosis and to validate the results obtained by other diagnostic modalities.

## Introduction

Appendicitis is caused by the inflammation, infection and clogging of the appendix^1^. The appendix is a tiny organ located in the lower abdomen connected to the large intestine. Appendicitis is extremely common in the United States, and at least 9% of the population experiences it^2^. The condition can cause internal puss, intense abdominal pain and block blood flow. Although younger children are susceptible to appendicitis, teenagers are the most common age group to experience it^3^. The appendix can rupture if the infection is not treated, and unwanted bacteria can emerge, leading to life-threatening conditions such as peritonitis and sepsis^1^.

**Fig. 1:**
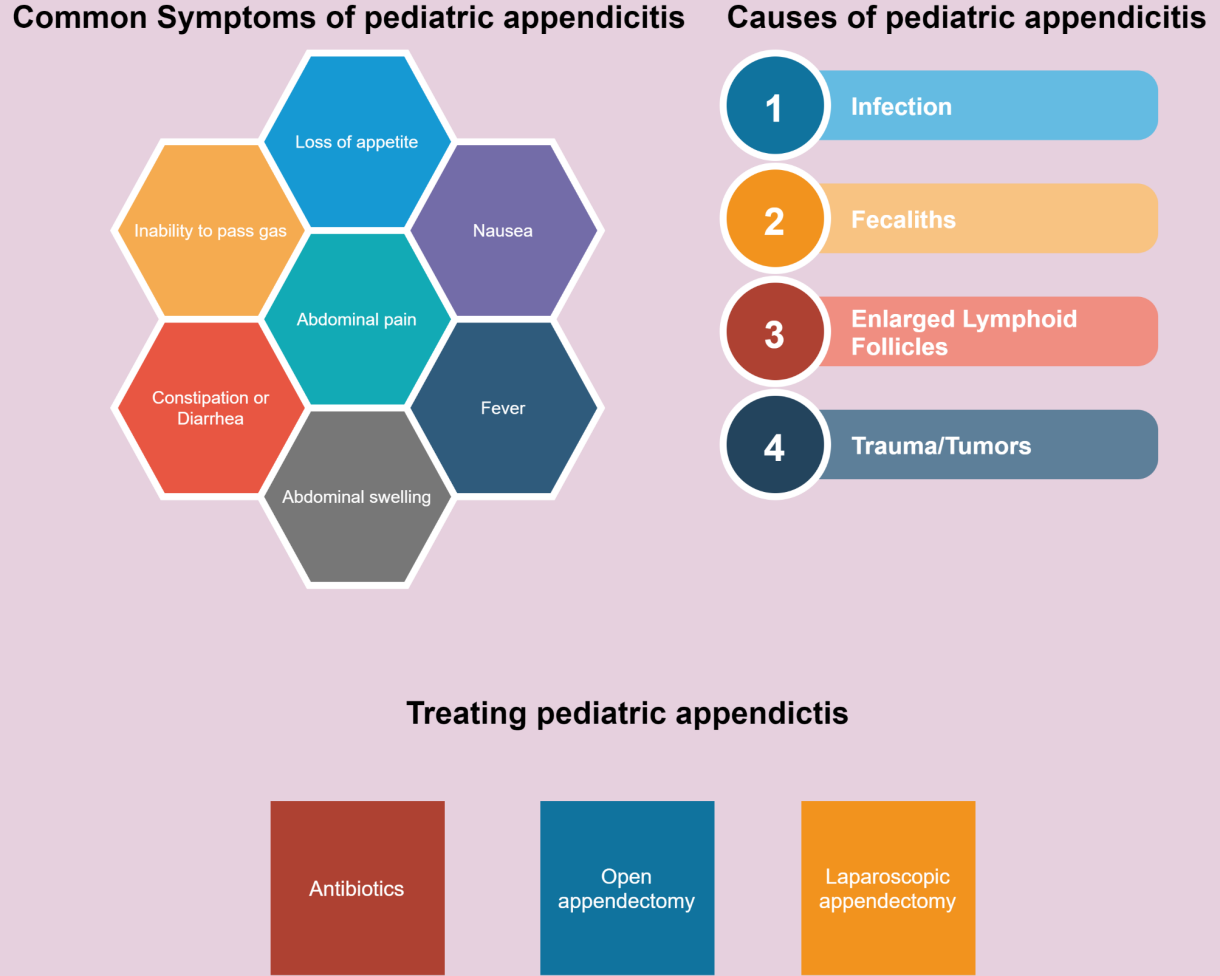
The common symptoms, causes and treatments for appendicitis in children.

Appendicitis is mainly caused by the obstruction in the opening of the appendix. Other contributing factors for this acute condition include abdominal infection, digestive tract infection, inflammatory bowel disease, and the growth of parasites inside the appendix^1^. Symptoms of this infection include lower abdominal pain, nausea, fever, loss of appetite, diarrhea, and a swollen belly. Most cases of appendicitis in children are treated surgically, either through laparoscopic or open surgery, with laparoscopic surgery being preferred due to its lower infection rate and shorter recovery time^4^. Mild cases can be treated without surgical treatment using antibiotics. Figure 1 summarizes various facts about pediatric appendicitis discussed above.

Appendicitis is diagnosed using a combination of laboratory and imaging tests^5^. Several blood and urine tests are conducted for effective diagnosis. It is also diagnosed using multiple imaging modalities such as abdominal X-rays, ultrasound, and computed tomography (CT) scans. The field of medicine extensively utilizes artificial intelligence (AI), a rapidly evolving and highly researched area of technology^6,7^. Computer systems make decisions based on various algorithms and statistical methodologies. There remains skepticism surrounding the utilization of AI frameworks in healthcare settings, primarily due to the intricate and highly challenging nature of interpreting these architectures^8^. However, a new branch of AI named Explainable AI (XAI) has emerged recently^8^. XAI assists demystifying the predictions made by the AI models using transparent, interpretable, and understandable techniques. The graphs and visualizations generated by these explainers enable the end user to comprehend the reasoning behind the decision-making processes of traditional black-box models. AI is increasingly employed to assist in diagnosis, prognosis, patient screening, and the efficient management of hospital systems^9^.

Multiple studies have been published that use machine learning (ML) to predict appendicitis in patients. Nie et al.^10^ used AI for differential diagnosis between acute appendicitis and Henoch-Schonlein purpura. 6965 patients, 53 markers, and five ML algorithms were considered in the study. The xgboost model obtained the best accuracy of 0.82. Lymphocyte ratio eosinophil ratio, eosinophil count, neutrophil ratio, and C-reactive protein were crucial markers identified in this study. In another research, machine learning was used to predict appendicitis in patients^11^. Among multiple algorithms, the random forest obtained optimal results with an accuracy of 83.75% for the dataset obtained from a public hospital. Predictive models were used to diagnose appendicitis in children in another study^12^. The dataset consisted of 430 children along with the results of clinical, laboratory and abdominal ultrasound tests. Three machine learning classifiers were used and a maximum area under precision recall curve of 0.94 was obtained for diagnosis. They also developed an online screening tool which could be easily accessible to the users. Aydin et al.^13^ used a machine learning approach to predict acute appendicitis in pediatric patients. The decision tree model was trained on the dataset consisting of 7244 patients. The classifier achieved an accuracy of 94.69%. Akbulut et al.^14^ designed an XAI framework to diagnose between perforated and non-perforated appendicitis. The research considered 1797 patients which were further divided into two groups. The Boruta algorithm was utilized to select the critical markers and the catboost classifier obtained an accuracy of 88.2%. Neutrophil, lymphocyte, platelet, age, and white blood cells were reported to be important features, according to Shapley additive values (SHAP).

There were a few research gaps in the existing studies. Most researchers did not make use of XAI techniques. In studies employing XAI techniques, their application was often limited to the utilization of SHAP explainer. Multiple XAI methods can be employed to enhance the interpretability of the model outputs. In contrast to many classifier pipelines developed for prediction that lacked optimization through hyperparameter tuning and extensive feature selection, this study undertook the optimization of several machine learning algorithms for predicting appendicitis among patients. The model outputs are further explained with multiple XAI tools. The contributions of this research are as follows:

- Six hyperparameter tuning techniques have been used to optimize the classifiers. They are: (a) Bayesian Optimization (b) Hybrid Bat Algorithm (c) Hybrid Self-adaptive Bat algorithm (d) Firefly Algorithm (e) Grid Search (f) Randomized Search.
- All the individual classifiers have been ensembled using a customized stacking model to form the “APPSTACK” model .
- Five XAI techniques make the predictions understandable, interpretable and transparent. They are (a) SHAP (b) LIME (Local Interpretable Model-agnostic Explanations) (c) QLattice (d) Eli5 (Explain like I am 5) (e) Anchor explainers. No other study has used five XAI methodologies to demystify pediatric appendicitis predictions. Medical professionals are now able to comprehend the results made by SHAP and LIME. We have also used Eli5, QLattice and Anchor which have been rarely used in medical literature.
- The pivotal markers elucidated through the explainers were subsequently scrutinized from a medical perspective for further validation.

## Materials and methods

### Dataset

This dataset was collected retrospectively from a group of children hospitalized for abdominal pain in “Children’s Hospital St. Hedwig”, Regensburg, Germany and the dataset is freely accessible on Mendeley too^15^. The dataset includes clinical markers, results of physical examination, and laboratory tests. The dataset contained test results of 782 patients along with 58 attributes. There were four potential target features in this dataset: “Presumptive Diagnosis”, “Diagnosis”, “Management” and “Severity”. This study focuses on designing ML/AI pipelines to accurately predict pediatric appendicitis. There were many missing values present in the collected data. The number of appendicitis cases was 465, and the number of non-appendicitis cases was 317. Table 1 presents a brief description of the features recorded in the dataset.

**Table 1:** Markers used to predict pediatric appendicitis^15^.

Marker	Description	Marker	Description	Marker	Description
1. Age	Patient’s age.	21. WBC_Count	White blood cell count.	41. Pathological_Lymph_Nodes	Whether lymph nodes have become enlarged?
2. Sex	Gender of the patient.	22. RBC_Count	Red blood cell count.	42. Lymph_Node_Location	Location of the lymph node.
3. Height	Patient’s height.	23. Hemoglobin	Hemoglobin percentage in blood.	43. Bowel_Wall_Thickening	Whether bowel walls have become thicker?
4. Weight	Patient’s weight.	24. RDW ( Red Cell Distribution Width)	A marker which indicates the size of red blood cells.	44. Ileus	Whether the patient is suffering from paralytic ileus?
5. Body mass index	It is an index which measures weight to height proportion.	25. Thrombocyte_Count	Platelets present in the blood.	45. Coprostasis	Whether fecal impaction in the colon exists?
6. Length of stay	The number of days admitted in the hospital.	26. Neutrophil_Percentage	Percentage of neutrophil in the blood.	46. Meteorism	Whether the intestine has excess gas?
7. Alvarado score	It is a score used to predict appendicitis in adults and children	27. Neutrophilia	Whether the patient is suffering from neutrophilia?	47. Enteritis	Whether enteritis exists?
8.Pediatric appendicitis score	It is a score which predicts appendicitis in children.	28. Segmented_Neutrophils	Matured neutrophils count.	48. Apendicolith	Whether fecalith exists in the appendix?
9. Peritonitis	Palpation which reveals a contraction of abdominal muscles, which is usually caused by inflammation.	29. CRP	C-reactive protein present in the body.	49. Perforation	Whether perforation exists in the appendix?
10. Migratory Pain	Localization of pain in the abdomen	30. Ketones_in_Urine	Ketones present in urine.	50. Appendicular_Abscess	Whether Appendiceal mass exists?
11. Lower_Right_Abd_Pain	Whether pain exists in the lower right part of the abdomen?	31. RBC_in_Urine	Red blood cells in urine.	51. Abscess_Location	Location where abscess exists.
12. Contralateral_Rebound_Tenderness	Whether pain exists in the contralateral part of the abdomen?	32. WBC_in_Urine	White blood cells in urine.	52. Conglomerate_of_Bowel_Loops	Any inflammation in small and large intestine ?
13. Ipsilateral_Rebound_Tenderness	Pain on the ipsilateral side occurs when pressure is released over the lower part of the abdomen.	33. US_Performed	Whether abdominal ultrasonography is performed?	53. Gynecological_Findings	Gynecolological abnormalities.
14. Coughing_Pain	Abdominal pain during coughing.	34. Appendix_on_US	Whether veriform appendix is detected during ultrasonography?	54. Ultrasound images	Ultrasound images of the appendix.
15. Psoas_Sign	Abdominal pain during hip extension.	35. Appendix_Diameter	Diameter of the appendix.	55. Diagnosis_Presumptive	Preliminary/ Initial diagnosis.
16. Nausea	Vomiting sensations.	36. Free_Fluids	Whether free fluids exist in the abdomen?	56. Diagnosis	Actual diagnosis
17. Loss_of_Appetite	Loss of appetite	37. Appendix_Wall_Layers	Whether appendix wall layers are normal?	57. Management	Managing appendicitis using various treatments.
18. Body_Temperature	Body temperature measured using thermometer	38. Target_Sign	A diagnosis sign based on axial images of the appendix.	58. Severity	Severity level of appendicitis.
19. Dysuria	Whether pain exists during urination?	39. Perfusion	Blood flowing to the appendix wall.		
20. Stool	Bowel types.	40. Surrounding_Tissue_Reaction	Whether inflammation exists outside the appendix?		

### Statistical analysis and data preprocessing

The initial data processing steps involved excluding features that were beyond the scope of our study, such as ‘ultrasound images,’ as well as target features like “preliminary diagnosis,” “management,” and “severity. The attributes “ketones in urine”, “RBC in urine” and “WBC in urine” were removed as these consisted of singleton values. Further, descriptive and inferential statistical analysis was performed on the data to identify key factors.

**Fig. 2:**
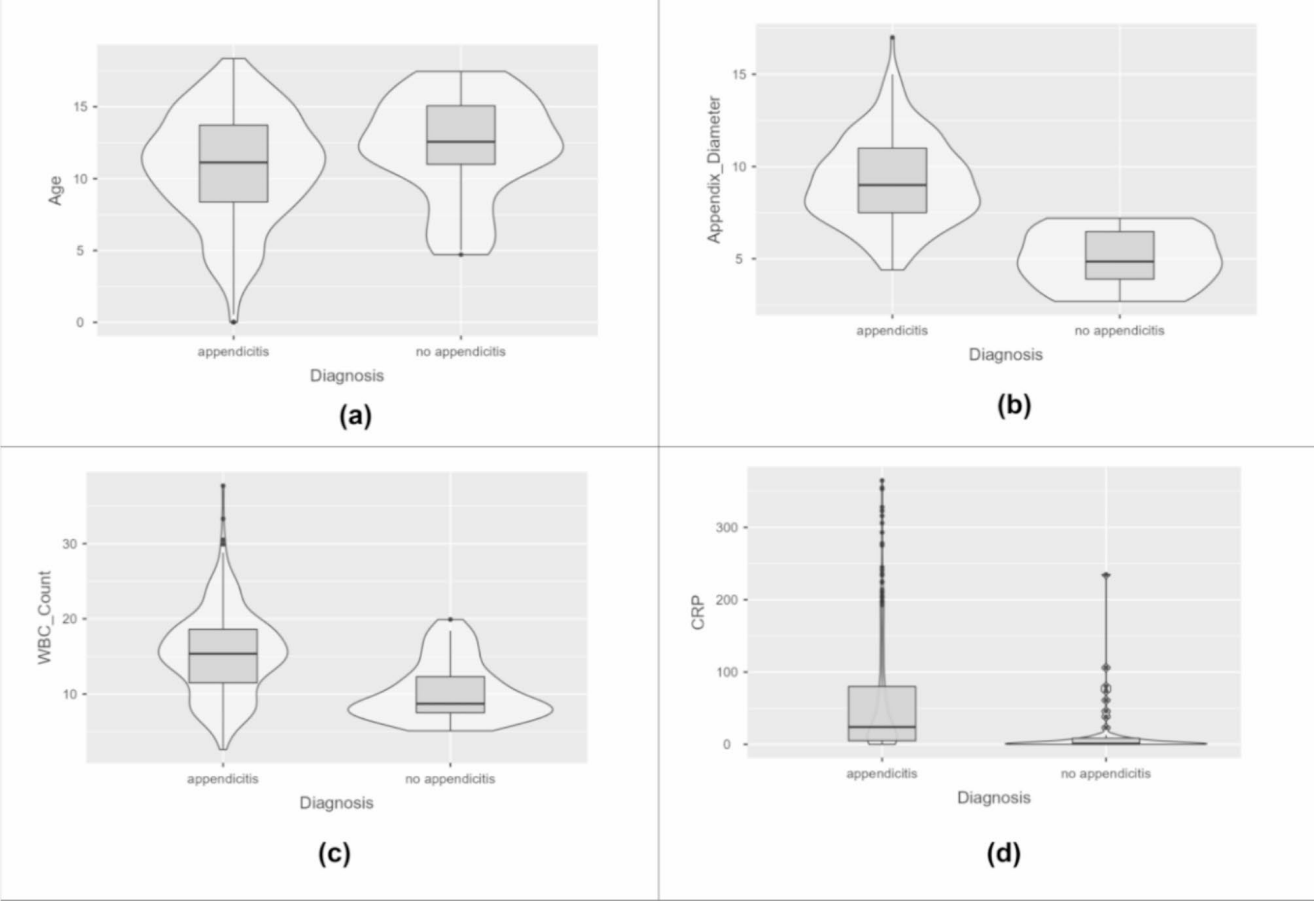
Violin plots to understand marker variation. (**a**) Age (**b**) Appendix diameter (**c**) WBC count (**d**) CRP.

Descriptive statistical parameters for the continuous attributes such as mean, mean, standard deviation, interquartile range, and range are described in Table 2. Violin plots for a few attributes have been depicted in Fig. 2 to identify marker variations. The mean age was higher in non-appendicitis patients. The plots depict higher appendix diameter, WBC count, and CRP levels among appendicitis patients.

**Table 2:** Descriptive statistical measures for a few continuous attributes.

	Diagnosis	Mean	Median	Standard deviation	Inter quartile range	Range
Age	Appendicitis	10.8	11.12	3.778	5.355	18.36
	No appendicitis	12.18	12.56	3.54	4.075	12.74
BMI	Appendicitis	18.33	17.56	4.315	5.133	29.21
	No appendicitis	19.34	18.83	4.511	5.76	18.05
Height	Appendicitis	145.23	147	21.871	29.5	137
	No appendicitis	152.35	156.5	17.982	19	79
Weight	Appendicitis	40.82	39	18.399	25.4	95.04
	No appendicitis	46.68	47	17.563	19.2	70.4
Appendix_Diameter	Appendicitis	9.19	9	2.317	3.5	12.6
	No appendicitis	4.99	4.85	1.598	2.575	4.5
Body_Temperature	Appendicitis	37.66	37.6	0.814	1.2	4.4
	No appendicitis	37.51	37.2	0.919	1.125	4
WBC_Count	Appendicitis	15.3	15.35	5.585	7.125	35.1
	No appendicitis	10.35	8.7	3.894	4.8	14.8
Neutrophil_Percentage	Appendicitis	78.31	80.4	11.161	12.1	68.2
	No appendicitis	66.85	69.45	15.76	27.375	53.4
RBC_Count	Appendicitis	4.78	4.79	0.377	0.49	2.32
	No appendicitis	4.79	4.82	0.41	0.555	1.68
Hemoglobin	Appendicitis	13.39	13.3	1.818	1.4	27.8
	No appendicitis	13.56	13.5	1.066	1.4	4.8
RDW	Appendicitis	13.69	12.9	7.198	0.8	75.4
	No appendicitis	12.99	12.9	0.742	0.95	3.1
Thrombocyte_Count	Appendicitis	290.37	277.5	77.251	99	610
	No appendicitis	280.95	276	73.563	94.5	272
CRP	Appendicitis	59.39	24	78.803	75	365
	No appendicitis	17.56	1	41.87	8.5	235
US_Number	Appendicitis	313.44	274	177.096	333	772
	No appendicitis	300.52	302	122.352	162.5	527

Inferential statistical testing was conducted using t-tests and chi square tests. Three types of t-tests have been used in this study. They are (a) Student’s t-test (b) Welch’s t-test (c) Mann Whitney U t-test^16^. If the obtained p-value is less than 0.001, we fail to reject the null hypothesis, indicating that the attribute is considered important for predicting appendicitis. The results of t-tests for some of the continuous attributes are described in Table 3. Attributes such as appendix diameter, WBC count, neutrophil percentage and CRP shows a significant marker between the two groups. Chi square tests were conducted on the categorical features as depicted in Table 4. Attributes such as length of stay, Alvarado score, pediatric appendicitis score, contralateral rebound tenderness, loss of appetite, neutrophilia and free fluids were more significant.

**Table 3:** Inferential statistical analysis of a few attributes using t- tests.

Attribute	Test type	*P*-value
Age	Student’s t	0.022
	Welch’s t	0.019
	Mann-Whitney U	0.016
BMI	Student’s t	0.147
	Welch’s t	0.165
	Mann-Whitney U	0.099
Height	Student’s t	0.038
	Welch’s t	0.019
	Mann-Whitney U	0.025
Weight	Student’s t	0.046
	Welch’s t	0.042
	Mann-Whitney U	0.026
Appendix_Diameter	Student’s t	< 0.001
	Welch’s t	< 0.001
	Mann-Whitney U	< 0.001
Body_Temperature	Student’s t	0.261
	Welch’s t	0.309
	Mann-Whitney U	0.135
WBC_Count	Student’s t	< 0.001
	Welch’s t	< 0.001
	Mann-Whitney U	< 0.001
Neutrophil_Percentage	Student’s t	< 0.001
	Welch’s t	< 0.001
	Mann-Whitney U	< 0.001
RBC_Count	Student’s t	0.936
	Welch’s t	0.94
	Mann-Whitney U	0.803
Hemoglobin	Student’s t	0.556
	Welch’s t	0.39
	Mann-Whitney U	0.221
RDW	Student’s t	0.525
	Welch’s t	0.109
	Mann-Whitney U	0.739
Thrombocyte_Count	Student’s t	0.453
	Welch’s t	0.439
	Mann-Whitney U	0.488
CRP	Student’s t	< 0.001
	Welch’s t	< 0.001
	Mann-Whitney U	< 0.001
US_Number	Student’s t	0.641
	Welch’s t	0.543
	Mann-Whitney U	0.913

**Table 4:** Inferential statistical analysis for a few attributes using chi-square tests.

Attribute	*p*-value
Sex	0.199
Length of stay	< 0.001
Alvarado score	< 0.001
Pediatric appendicitis score	< 0.001
Migratory Pain	0.005
Lower_Right_Abd_Pain	0.004
Contralateral_Rebound_Tenderness	< 0.001
Coughing_Pain	0.014
Nausea	0.378
Loss_of_Appetite	< 0.001
Neutrophilia	< 0.001
Dysuria	0.383
Free_Fluids	< 0.001

The missing values were removed using the respective median of the attributes. This is an effective null-value-removal technique in machine learning^17^. Data scaling is often recommended to prevent potential biases^18^. The Min-Max normalization technique was used to scale the feature values between 0 and 1. Encoding categorical variables is crucial to prevent model overfitting. In this study, we utilized the one-hot encoding technique for variable encoding^19^. Additionally, this dataset exhibited a slight issue with the target class being imbalanced. Without data balancing, classifiers may exhibit a tendency to prioritize the majority class, potentially leading to biased predictions and overlooking the significance of the minority class^20^. In this study, the target class was balanced using the Borderline-SMOTE technique^21^. This oversampling technique creates new samples of the minority class that are similar to the existing instances using the k means algorithm. After performing data balancing, the dataset was split into training (80%) and testing (20%) subsets.

### Customized STACK model, XAI techniques and hyperparameter optimization

**Fig. 3:**
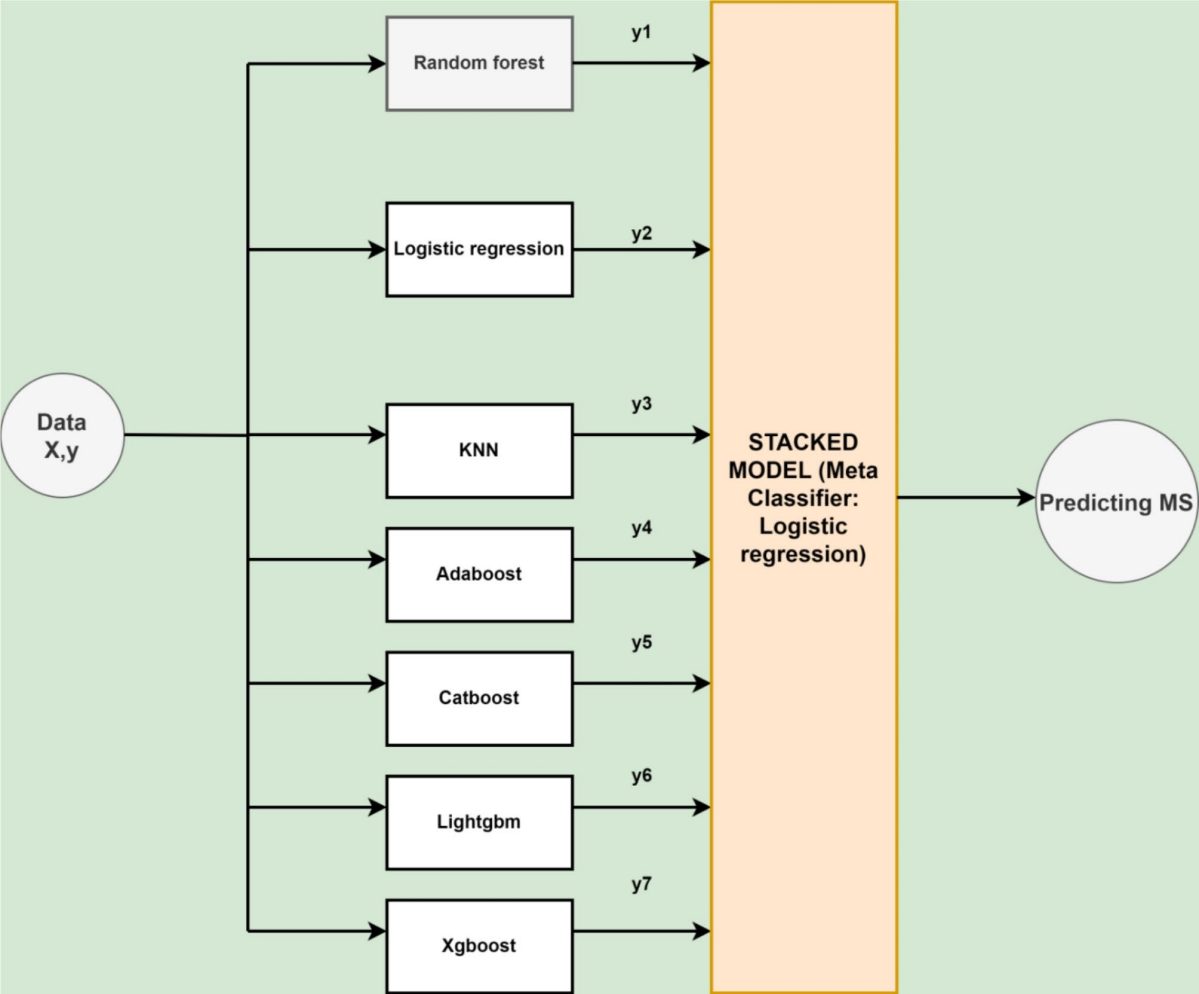
The architecture of the proposed “APPSTACK” model created using eight baseline classifiers.

Several classifiers have been ensembled using the stacking methodology in this study. Stacking improves the predictive performance by combining the prediction power of various classifiers using a meta-learner^22^. Stacking is useful as it can identify various trends in the information that other algorithms might overlook. Combining distinct models can also help minimize overfitting, specifically when the predicted biases or errors vary across different data subsets. The proposed architecture of the APPSTACK model to predict appendicitis is described in Fig. 3.

We have also used five XAI techniques to make the predictions of the algorithms interpretable. The algorithms are briefly explained below:

- **SHAP**: SHAP is an approach based on game theory that can be employed to comprehend the outcomes of any classifier^23^. It links best allocation of credit to local explanations by employing game theory’s classic Shapley values and their associated modifications. The module is directly available in Python. The values of SHAP allow the outcome value to be distributed among the attributes for a particular estimation. Every attribute is assigned a SHAP value, which indicates how much it contributes to the definitive foresight. SHAP supports consistency and fairness. Consistency implies that if the value of an attribute remains constant, its impact must stay consistent. Fairness means one’s contribution are equally split among the characteristics. It can also be applied to most algorithms since it is model agnostic. There are three general steps for interpretation using SHAP. In the beginning, SHAP values are computed for every feature. The values then undergo interpretation to understand the effectiveness of every attribute. In the final step, the interpretations are visualized using various plots.

- **LIME**: LIME was introduced by Marco Riberio in the year 2016^24^. It supports a wide range of ML algorithms since it is model agnostic. However, it does not make global interpretations. Using local interpretations, we can use the LIME model to interpret each patient-level output. There are a series of steps followed by the LIME model to explain the predictions. Initially, the data points are distributed using a normal distribution. Y coordinates are then predicted using the given ML algorithm. RBF kernel is then utilized to assign weights. In the last step, ridge regression model is trained on the weighted dataset. The predictions are then described using various plots and graphs. The interpretable model which the LIME produces is not complicated to understand. LIME operates by perturbing the input features of an individual data instance and observing how these changes affect the predictions of the underlying machine learning model. To roughly represent the values of the intricate classifier in close proximity, it creates an explainable linear model. LIME collects altered cases in the vicinity of the unique instance and employs them for learning the comprehensible framework. The process of sampling is frequently accomplished through the addition of unpredictability or by ensuring minor modifications to the initial attributes. The weighing of the instances is then determined by how close they are to the initial instance.
- **Eli5**: Eli5 is a library in python which could be used to demystify the algorithm’s predictions^25^. It supports a variety of algorithms in ML. Eli5 has several advantages. It can handle minor consistencies effectively. It also supports code reusability. Eli5 is known to handle both global and local interpretations.
- **QLattice**: QLattice was created and developed by ‘Abzu’^26^. The module is based on the concept of symbolic regression. The module accepts information of both numerical and categorical types. The model explanation is made using QGraphs. QGraphs contain activation function, edges and nodes. Activation function is used to transform the output, edges connect the nodes and each attribute is represented as a node.
- **Anchor**: Anchors utilizes the concept of ‘conditions’ and ‘rules’ to explain predictions^27^. The strength of the anchor is quantized using two metrics: Coverage and Precision. Coverage is the total amount of cases that use the exact same condition for estimation. The accuracy of the explanations is measured using Precision.

Hyperparameter tuning is necessary in machine learning to increase the accuracy of the data. Search techniques are utilized to find each algorithm’s best set of hyperparameters. In this study, we have utilized six techniques to identify the critical parameters. They are as follows:

- **Bayesian Optimization**: This method uses Bayes’ theorem to search the hyperparameters^28^. After defining the search space, an acquisition function balances the exploration and exploitation phase during the search process.
- **Hybrid Bat Algorithm**: This metaheuristic algorithm uses the Bat Algorithm with other techniques to perform accurately^29^. The algorithm has various phases: initialization, echolocation and movement, frequency adjustment, and iteration.
- **Hybrid Self-adaptive Bat Algorithm**: In this technique, the Bat Algorithm is modified using self-adaption algorithms, which modify its parameters during the optimization phase^30^. This enables the algorithm to continually modify its parameters based on the features of the optimization problem being resolved, enhancing its efficiency.
- **Firefly Algorithm**: This algorithm is based on the behavior of fireflies^31^. They emit light with various intensities depending on the strength of the insect. The algorithm comprises initialization, evaluation, movement, updating brightness, and iterative movements.
- **Grid Search**: A grid of hyperparameters is defined in grid search. Each combination in the grid is separately analyzed and evaluated^32^. The various steps in this method are: Defining the hyperparameter grid, model training/evaluation, selecting the best hyperparameters and validating the model.
- **Randomized Search**: Randomized search searches a few combinations randomly instead of doing an exhaustive search^33^. This technique is faster than the grid search technique. However, the grid search is more effective since it searches all possible combinations. The various steps in this technique are: defining the hyperparameter distributions, random sampling, model training and evaluation, selecting the best hyperparameters and model validation.

**Fig. 4:**
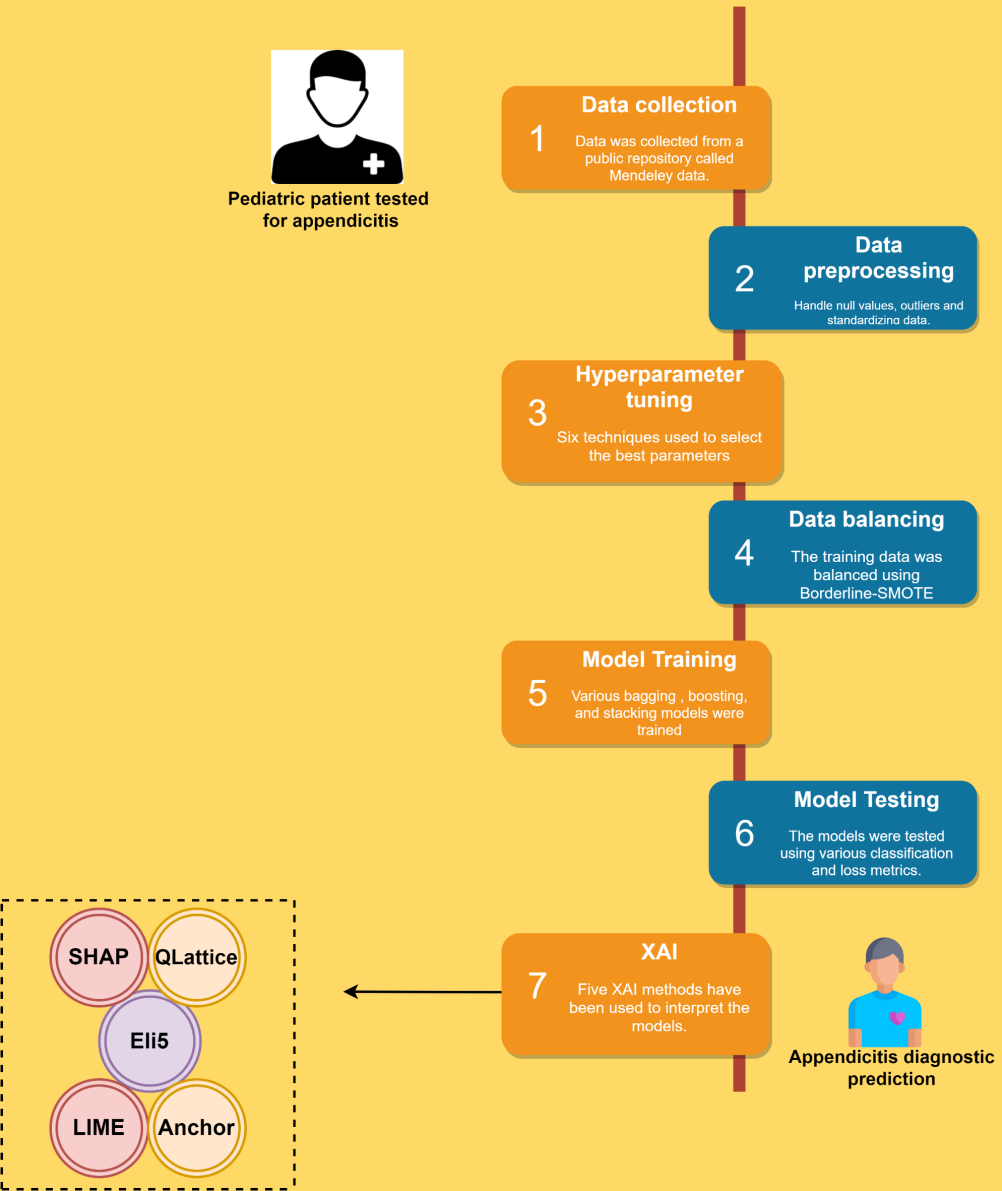
Machine learning pipeline used in this study to detect appendicitis and interpret the models.

The machine learning pipeline utilized in this study is described in Fig. 4.

## Results

**Fig. 5:**
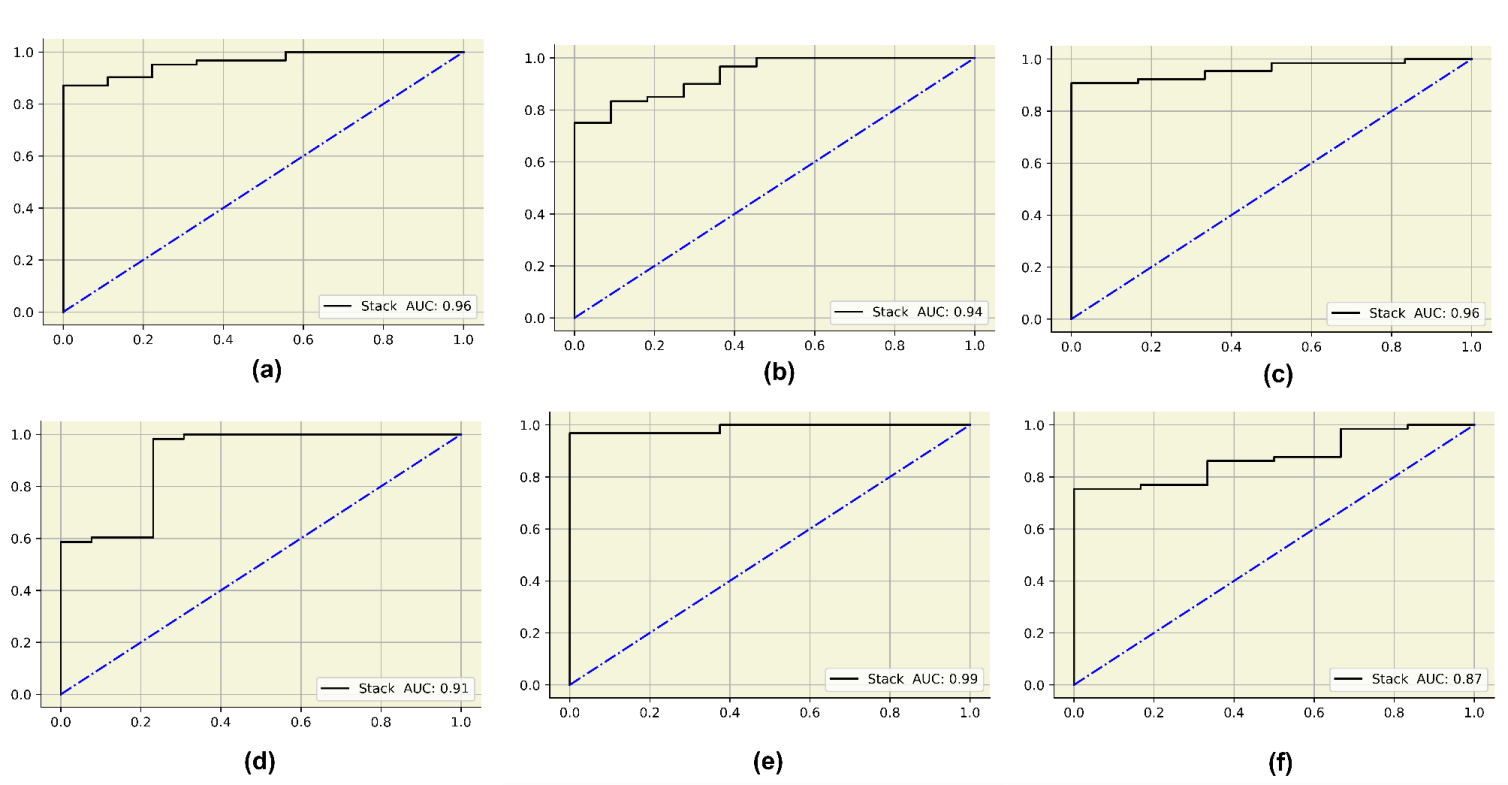
AUC curves for the final APPSTACK model. (**a**) Grid search (**b**) Randomized search (**c**) Hybrid bat algorithm (**d**) Hybrid bat self-adaptive algorithm (**e**) Firefly algorithm (**f**) Bayesian optimization algorithm.

**Fig. 6:**
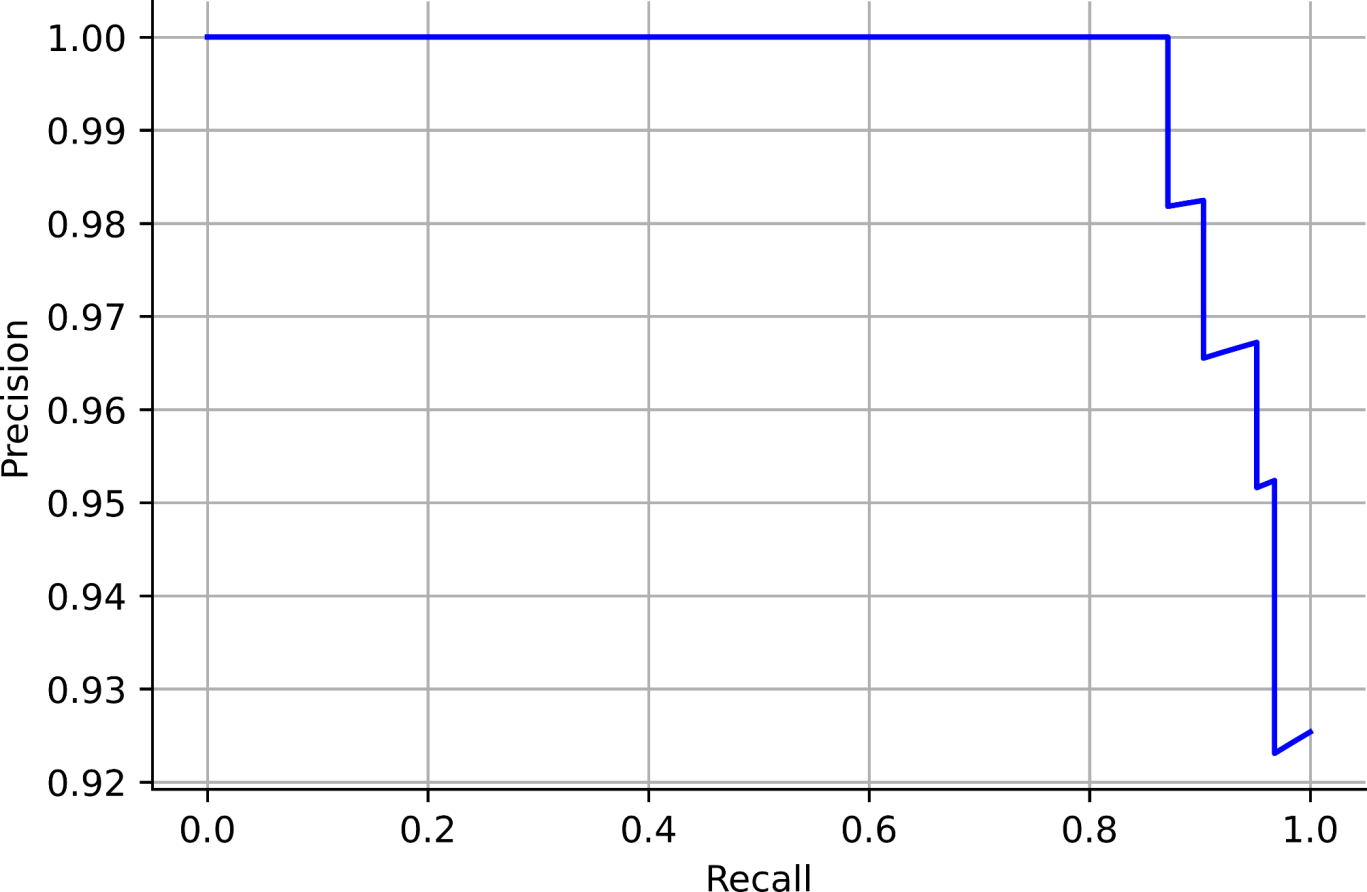
(a) Precision-Recall curve for APPSTACK (Hybrid bat algorithm).

In this study, six searching techniques have been utilized to find the optimal hyperparameters. When the grid search was used, the random forest, catboost and xgboost obtained an accuracy of 94%. The APPSTACK obtained an accuracy of 92%. A F1-score of 87% was obtained by the random forest when grid search was used. When the randomized search was used, the xgboost obtained an accuracy of 94% and the APPSTACK obtained an accuracy of 92%. An F1-score of 87% was obtained by the xgboost when randomized search was used. When the hybrid Bat Algorithm was used, the xgboost and adaboost obtained an accuracy of 96% and the APPSTACK obtained an accuracy of 94%. An F1-score of 86% was obtained by the adaboost algorithm. When the Hybrid Self-adaptive Algorithm was used, an accuracy of 95% was obtained by the adaboost model and the APPSTACK obtained an accuracy of 93%. A F1-score of 89% was obtained by the adaboost, catboost and xgboost model. When the Firefly Searching method was utilized, xgboost obtained an accuracy of 97% and the APPSTACK obtained an accuracy of 93%. The xgboost also obtained the highest F1-score of 93%. Bayesian Optimization did not perform well compared to the other three algorithms. An accuracy of 92% was obtained by the catboost algorithm when Bayesian Optimization was utilized. The APPSTACK obtained an accuracy of 92%. The precision and recall obtained were poor when using this searching technique. The classification results are detailed in Table 5. Among the searching techniques, Hybrid Bat Algorithm performed the best since it obtained an accuracy of 94% for the APPSTACK model. The hyperparameters chosen by all the algorithms for the hybrid bat algorithm searching technique are depicted in Table 6. The AUC curves for all the APPSTACK models are depicted in Fig. 5. The precision-recall curve obtained by the APPSTACK model for the hybrid bat algorithm is depicted in Fig. 6. It can be inferred that the number of false positive and false negative cases were very few and the predictions were made accurately. Most of the algorithms obtained good results due to the use of hyperparameter, data balancing and other important data preprocessing techniques.

**Table 5:** Pediatric appendicitis classification results.

Grid search									
Algorithm	Accuracy (%)	Precision (%)	Recall (%)	F1-score (%)	AUC	Hamming loss	Jaccard score	Log loss	Mathew’s correlation coefficient
**Random forest**	94	87	87	87	0.97	0.05	0.93	1.94	0.74
**Logistic regression**	93	85	82	83	0.95	0.07	0.92	2.43	0.66
**Decision tree**	86	68	68	68	0.73	0.14	0.85	4.86	0.36
**KNN**	87	72	78	75	0.78	0.12	0.86	4.37	0.50
**Adaboost**	92	83	76	79	0.87	0.08	0.90	2.91	0.58
**Catboost**	94	91	83	86	0.97	0.05	0.93	1.94	0.72
**Lightgbm**	90	79	71	74	0.9	0.09	0.09	3.40	0.49
**Xgboost**	94	87	87	87	0.94	0.05	0.93	1.94	0.72
**APPSTACK**	92	81	81	81	0.96	0.08	0.90	2.91	0.61
**Randomized search**									
**Algorithm**	**Accuracy (%)**	**Precision (%)**	**Recall (%)**	**F1-score (%)**	**AUC**	**Hamming loss**	**Jaccard score**	**Log loss**	**Mathew’s correlation coefficient**
**Random forest**	92	89	76	81	0.94	0.08	0.90	2.91	0.64
**Logistic regression**	92	86	80	83	0.95	0.08	0.90	2.91	0.65
**Decision tree**	77	61	64	62	0.7	0.22	0.75	7.78	0.25
**KNN**	68	66	81	63	0.9	0.32	0.61	11.18	0.44
**Adaboost**	87	76	78	77	0.88	0.12	0.85	4.37	0.53
**Catboost**	90	82	79	80	0.95	0.09	0.89	3.40	0.60
**Lightgbm**	90	82	79	80	0.94	0.09	0.89	3.40	0.60
**Xgboost**	94	97	82	87	0.95	0.99	0.93	1.94	0.77
**APPSTACK**	92	86	80	83	0.94	0.08	0.90	2.91	0.65
**Hybrid bat algorithm (HBA)**									
**Algorithm**	**Accuracy (%)**	**Precision (%)**	**Recall (%)**	**F1-score (%)**	**AUC**	**Hamming loss**	**Jaccard score**	**Log loss**	**Mathew’s correlation coefficient**
**Random forest**	94	97	66	73	0.94	0.04	0.91	1.94	0.56
**Logistic regression**	94	81	81	81	0.91	0.05	0.94	1.94	0.63
**Decision tree**	83	60	69	62	0.83	0.16	0.82	5.83	0.26
**KNN**	80	45	43	44	0.85	0.19	0.80	5.83	0.26
**Adaboost**	96	81	83	86	0.94	0.04	0.95	1.45	0.70
**Catboost**	93	78	82	79	0.94	0.07	0.92	2.43	0.57
**Lightgbm**	92	73	73	73	0.95	0.08	0.91	2.91	0.45
**Xgboost**	96	97	75	83	0.91	0.04	0.95	1.45	0.69
**APPSTACK**	94	85	74	78	0.96	0.05	0.94	1.94	0.58
**Hybrid self-adaptive bat algorithm**									
**Algorithm**	**Accuracy (%)**	**Precision (%)**	**Recall (%)**	**F1-score (%)**	**AUC**	**Hamming loss**	**Jaccard score**	**Log loss**	**Mathew’s correlation coefficient**
**Random forest**	93	92	84	88	0.95	0.07	0.91	2.43	0.75
**Logistic regression**	87	79	80	79	0.93	0.15	0.82	4.37	0.58
**Decision tree**	85	75	73	73	0.85	0.15	0.82	5.35	0.46
**KNN**	75	65	72	66	0.75	0.25	0.70	8.75	0.37
**Adaboost**	95	97	85	89	0.93	0.05	0.72	1.94	0.80
**Catboost**	94	97	85	89	0.95	0.05	0.93	1.94	0.80
**Lightgbm**	92	93	92	84	0.87	0.08	0.91	2.43	0.57
**Xgboost**	94	97	85	89	0.95	0.05	0.93	1.94	0.80
**APPSTACK**	93	92	84	87	0.91	0.07	0.91	2.43	0.75
**Firefly algorithm**									
**Algorithm**	**Accuracy (%)**	**Precision (%)**	**Recall (%)**	**F1-score (%)**	**AUC (%)**	**Hamming loss (%)**	**Jaccard score (%)**	**Log loss (%)**	**Mathew’s correlation coefficient (%)**
**Random forest**	93	81	91	85	0.98	0.07	0.92	2.43	0.71
**Logistic regression**	83	67	80	70	0.89	0.16	0.81	5.83	0.44
**Decision tree**	90	75	78	77	0.82	0.09	0.89	3.40	0.53
**KNN**	81	65	78	68	0.81	0.19	0.78	6.81	0.40
**Adaboost**	86	70	81	73	0.96	0.14	0.85	4.86	0.49
**Catboost**	89	74	88	78	0.97	0.11	0.87	3.89	0.61
**Lightgbm**	89	74	88	78	0.98	0.11	0.875	3.91	0.60
**Xgboost**	97	93	93	93	0.98	0.02	0.96	0.97	0.86
**APPSTACK**	93	81	96	86	0.99	0.07	0.92	2.43	0.75
**Bayesian optimization**									
**Algorithm**	**Accuracy (%)**	**Precision (%)**	**Recall (%)**	**F1-score (%)**	**AUC**	**Hamming loss**	**Jaccard score**	**Log loss**	**Mathew’s correlation coefficient**
**Random forest**	89	59	57	57	0.92	0.11	0.88	3.89	0.14
**Logistic regression**	86	63	70	65	0.88	0.14	0.85	4.86	0.31
**Decision tree**	78	58	58	58	0.71	0.11	0.81	3.71	0.29
**KNN**	80	55	59	55	0.59	0.19	0.79	6.81	0.13
**Adaboost**	90	67	64	66	0.92	0.09	0.89	3.40	0.31
**Catboost**	92	73	73	73	0.89	0.08	0.91	2.91	0.45
**Lightgbm**	89	64	64	64	0.92	0.11	0.88	3.87	0.27
**Xgboost**	92	72	65	68	0.91	0.08	0.91	2.91	0.36
**APPSTACK**	87	61	63	62	0.87	0.12	0.86	4.37	0.23

**Table 6:** Hyperparameters chosen by algorithms for the hybrid bat algorithm searching technique.

Algorithm	Hyperparameters
Random forest	{‘n_estimators’: 60, ‘max_depth’: 18, ‘min_samples_split’: 10, ‘max_features’: ‘log2’}
Logistic regression	{‘penalty’: ‘l2’, ‘C’: 1000}
Decision tree	{‘criterion’: ‘gini’, ‘max_depth’: 10, ‘min_samples_split’: 10, ‘splitter’: ‘best’, ‘min_samples_leaf’: 1, ‘max_features’: ‘auto’}
KNN	{‘n_neighbors’: 1}
Adaboost	{‘n_estimators’: 1000, ‘learning_rate’: 1.0}
Catboost	{‘depth’: 3, ‘iterations’: 250, ‘learning_rate’: 0.03, ‘l2_leaf_reg’: 5, ‘border_count’: 10}
Lightgbm	{‘num_leaves’: 127, ‘reg_alpha’: 0.5, ‘min_data_in_leaf’: 50, ‘lambda_l1’: 0, ‘lambda_l2’: 0}
Xgboost	{‘learning_rate’: 0.1, ‘max_depth’: 8, ‘min_child_weight’: 1, ‘gamma’: 0.1, ‘colsample_bytree’: 0.3}
APPSTACK	(random_state = 42, max_iter = 9000, use_probas = True, average_probas = False

**Fig. 7:**
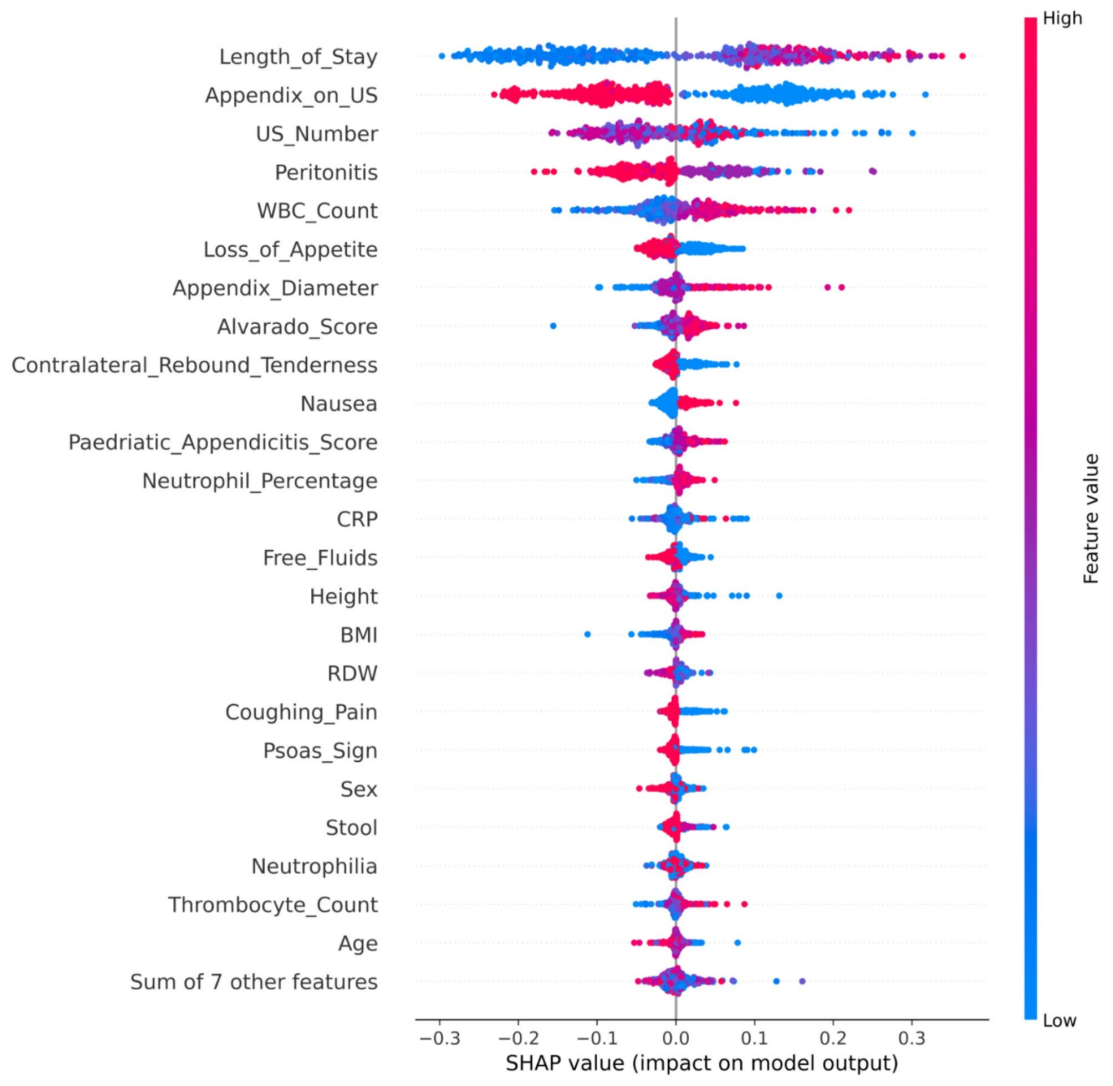
SHAP Beeswarm plot to decipher pediatric appendicitis prediction.

**Fig. 8:**
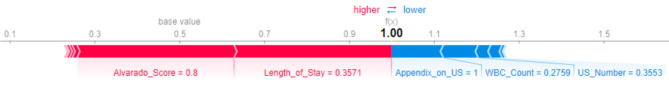
SHAP force plot for an individual appendicitis patient.

**Fig. 9:**
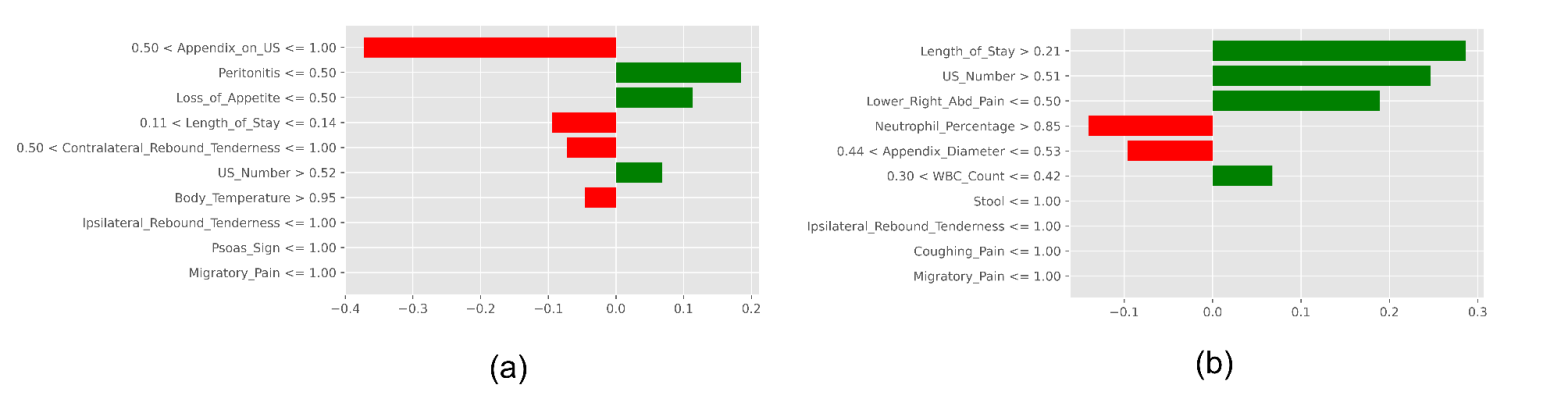
LIME model to decipher model predictions. (a) Appendicitis negative diagnosis (b) Appendicitis positive diagnosis.

**Fig. 10:**
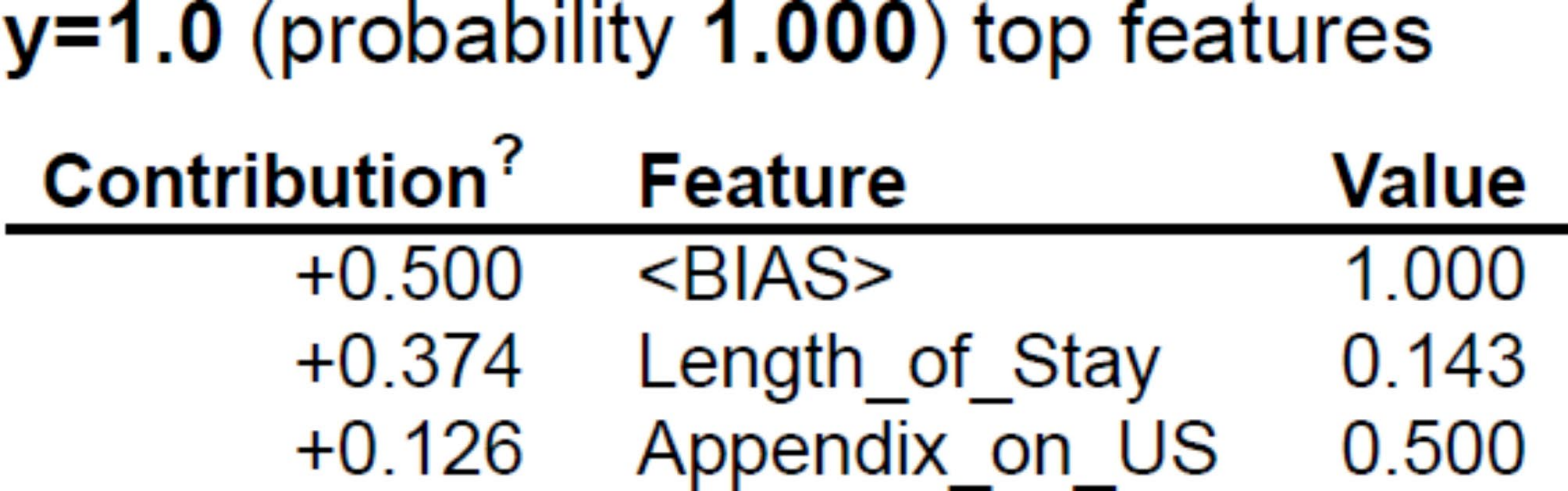
Eli5 technique to understand crucial parameters in pediatric appendicitis prediction.

**Fig. 11:**
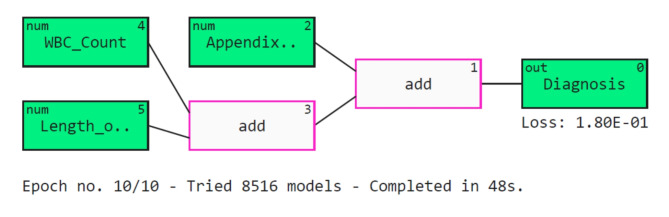
QGraphs to understand important markers in pediatric appendicitis prediction.

Further, five explainers were used to explain the predictions. The APPSTACK model with the hybrid bat algorithm was used for further analysis. The beeswarm plot for the SHAP explainer is depicted in Fig. 7. The markers are organized in the descending order of their significance. Hence, length of stay, appendix on ultrasonography, peritonitis, white blood cell count, loss of appetite and appendix diameter were the crucial attributes. Further, a vertical plane separates the two classes. The color-coding scheme is as follows: Blue signifies lower values and red signifies higher values. When the length of stay is more, there is a higher chance of appendicitis diagnosis. If the appendix is clearly visible during the ultrasonography exam, there was a higher chance that the patient did not suffer from appendicitis. Local interpretations (individual patient prediction) can be made in SHAP using a force plot. A sample force plot is depicted in Fig. 8. From the graph, it can be inferred that attributes such as Alvarado score and length of stay are pushing the predictions towards a positive appendicitis diagnostic prediction. LIME predictions for a non-appendicitis patient are made in Fig. 9(a). Attributes such as length of stay, appendix on ultrasonography and contralateral rebound tenderness are pointing towards the same outcome. LIME prediction for an appendicitis patient is made in Fig. 9(b). Attributes such as Length of stay and lower right abdominal pain are pointing towards the same. Parameters with higher weights are given more preference in LIME. Eli5 was the next explainer utilized and the interpretations made by it are detailed in Fig. 10. It can be inferred that length of stay and appendix on ultrasonography were the most important markers. The QGraph generated by the QLattice model is depicted in Fig. 11. According to them, the best markers are White blood cell count, length of stay and appendix on ultrasonography. In this study, the QLattice made use of the “addition” activation function. The last explainer used is anchor. It consists of a condition and is measured by its precision and coverage. Precision is the accuracy and coverage are the range of a particular condition. Anchor explanations for appendicitis positive/negative patient are made in Table 7. The most important markers are length of stay, appendix on ultrasonography, white blood cells and appendix diameter.

Five XAI techniques have been used and according to them, the critical variables are length of stay, appendix on ultrasonography, white blood cells and appendix diameter. These markers can be used to predict appendicitis in pediatric patients.

**Table 7:** Explanations made by Anchor in diagnosing COVID-19.

Patient Type	Anchor	Precision	Recall
Not appendicitis	Appendix_on_US < = 0.50 AND US_Number < = 0.29	0.84	0.20
Not appendicitis	Length_of_Stay < = 0.14 AND WBC_Count < = 0.33	0.75	0.36
Not appendicitis	Length_of_Stay < = 0.11 AND Appendix_on_US > 0.50	0.83	0.31
Appendicitis	Length_of_Stay > 0.20 AND WBC_Count > 0.43	0.94	0.11
Appendicitis	Length_of_Stay > 0.11 AND Appendix_Diameter > 0.53	1	0.14
Appendicitis	Length_of_Stay > 0.11 AND Appendix_on_US < = 0.50	0.96	0.33

## Discussion

In this study, multiple classifiers were utilized to diagnose appendicitis in pediatric patients. To optimize the algorithms, five different hyperparameter tuning techniques were used. Among them, the Hybrid Bat Algorithm proved to be superior over the other searching techniques. The customized stack algorithm “APPSTACK” obtained an accuracy of 94%. Important markers were identified using five different XAI techniques. According to them, the crucial attributes are length of stay, appendix on ultrasonography, white blood cells and appendix diameter.

In this research, when the length of stay (hospital admissions) was more, there was a higher chance of getting diagnosed with appendicitis. If the appendix was clearly visible during ultrasonography, the probability of appendicitis was very less. Peritonitis (Inflammation of the abdomen) was observed in both appendicitis and non-appendicitis patients. White blood cell count was higher in pediatric appendicitis patients. In this research, the appendix diameter was higher in appendicitis patients. Nausea was also observed in the appendicitis cohort. Higher Alvarado score and appendicitis score were also observed in the positive diagnosis cohort. Lastly, higher neutrophil percentage in the appendicitis cohort. Many of these trends have also been observed in other similar appendicitis studies^34–36^. The variation in these markers is accurately identified by the classifiers to make precise diagnosis which could aid the doctors and other healthcare personnel.

A few researchers have used ML to diagnose appendicitis. Nie et al.^10^ used AI to diagnose appendicitis in children. Five ML models and 53 markers were utilized in this research which consisted of 6965 patients. Xgboost obtained the highest AUC of 0.895. Mijwil et al.^11^ used ML techniques to diagnose appendicitis. The number of patients considered was 625 and the random forest was able to obtain a maximum accuracy of 83.75%. In another research, ML algorithms were used to accurately predict appendicitis^12^. An AUC of 0.96 was obtained by the random forest and gradient boosting machine model. The comparison of our model with similar studies is made in Table 8. No previous studies have employed five Explainable Artificial Intelligence (XAI) techniques for predicting appendicitis in pediatric patients.

**Table 8:** A few studies which use AI to predict appendicitis.

Author	Dataset size	ML models used	Maximum results	XAI
Nie et al. [10]	6965 patients	Five models	AUC-0.895	Xgoost feature ranking and SHAP
Mijwil et al. [11]	625 patients	Seven models	Accuracy – 83.75%	-
Marvinkevics et al. [12]	430 patients	Three models	AUC- 0.96	-
Aydin et al. [13]	7244 patients	Six models	AUC-0.93, Accuracy − 94%	-
Akbulut et al. [14]	1797 patients	Various models	Accuracy – 92%	-
This study	782 patients	Various models + APPSTACK (customized ensemble)	Accuracy-94% AUC-0.96	SHAP, LIME, Eli5, QLattice and Anchor

There were a few limitations in this research. The patient data chosen in this research consisted of only 782 cases. In this research, deep learning algorithms were not employed due to their preference for large-scale datasets. Cloud-based systems were not utilized in this study. Although cloud infrastructures can enhance data accessibility and security, they were not employed in this research.

## Conclusion

Explainable artificial intelligence algorithms were used to interpret the appendicitis predictions made by the customized APPSTACK model in this research. Initially, the dataset was subjected to statistical analysis to gain more inferences on the data. Further five searching techniques namely: Hybrid Bat Algorithm, Self-adaptive Bat Algorithm, Firefly Algorithm, Randomized Search and Grid Search were used to find the optimal hyperparameters. A customized ensemble algorithm (APPSTACK) was designed using the stacking methodology. The algorithms were trained and tested for all the five searching techniques. A maximum accuracy of 94% was obtained when the Hybrid Bat Algorithm was used. Five explainers were utilized to decipher the results and understand the critical parameters. According to them, the critical variables are length of stay, appendix on ultrasonography, white blood cells and appendix diameter. The variations in these markers can be thoroughly analyzed to decipher the diagnoses determined by supervised learning algorithms. Through this analysis, the models have the potential to significantly enhance the efficiency in the healthcare sector by assisting doctors and medical professionals in accurately detecting pediatric appendicitis and distinguishing it from other abdominal illnesses.

This study focuses on supervised learning. Federated learning, unsupervised learning and reinforcement learning algorithms could be explored in the future. Future work can also consist of building a user-friendly interface for easy diagnostic prediction. The applications can be used real time in several hospitals and medical facilities. Cryptography and steganography algorithms can be used to secure the data. Data can be collected from various hospitals and combined so that the models become more reliable and generalizable.

## Data Availability

Data will be made available by Dr. Krishnaraj Chadaga on prior request.
