# Author Correction: An interpretable and transparent machine learning framework for appendicitis detection in pediatric patients

**DOI:** 10.1038/s41598-025-86494-x

**Published:** 2025-01-22

**Authors:** Krishnaraj Chadaga, Varada Khanna, Srikanth Prabhu, Niranjana Sampathila, Rajagopala Chadaga, Shashikiran Umakanth, Devadas Bhat, K. S. Swathi, Radhika Kamath

**Affiliations:** 1 Department of Computer Science and Engineering, Manipal Institute of Technology, Manipal Academy of Higher Education, Manipal, 576104 Karnataka India; 2 Department of Biostatistics, Yale School of Public Health, Yale University, New Haven, Connecticut, 06510 USA; 3 Department of Biomedical Engineering, Manipal Institute of Technology, Manipal Academy of Higher Education, Manipal, 576104 Karnataka India; 4 Department of Mechanical and Industrial Engineering, Manipal Institute of Technology, Manipal Academy of Higher Education, Manipal, 576104 Karnataka India; 5 Department of Medicine, Dr. TMA Pai Hospital, Manipal Academy of Higher Education, Manipal, 576104 Karnataka India; 6 Department of Social and Health Innovation, Prasanna School of Public Health, Manipal Academy of Higher Education, Manipal, 576104 Karnataka India

Correction to: *Scientific Reports* 10.1038/s41598-024-75896-y, published online 18 October 2024

In the original version of this Article, Shashikiran Umakanth was incorrectly affiliated with ‘Department of Computer Science and Engineering, Manipal Institute of Technology, Manipal Academy of Higher Education, Manipal, 576104, Karnataka, India’, K.S. Swathi was incorrectly affiliated with 'Department of Medicine, Dr. TMA Pai Hospital, Manipal Academy of Higher Education, Manipal, 576104, Karnataka, India’ and Radhika Kamath was incorrectly affiliated with ‘Department of Social and Health Innovation, Prasanna School of Public Health, Manipal Academy of Higher Education, Manipal, 576104, Karnataka, India.'

Consequently, the correct affiliations are as follows:

Shashikiran Umakanth

5. Department of Medicine, Dr. TMA Pai Hospital, Manipal Academy of Higher Education, Manipal, 576104, Karnataka, India

K.S. Swathi

6. Department of Social and Health Innovation, Prasanna School of Public Health, Manipal Academy of Higher Education, Manipal, 576104, Karnataka, India

Radhika Kamath

1. Department of Computer Science and Engineering, Manipal Institute of Technology, Manipal Academy of Higher Education, Manipal, 576104, Karnataka, India

The original Article has been corrected.

